# Development of a quantitative PCR assay to detect *Gambierdiscus holmesii*, a ciguatoxin producing species from Australian waters

**DOI:** 10.1371/journal.pone.0355213

**Published:** 2026-08-03

**Authors:** Caroline Dornelles de Azevedo, Greta Gaiani, Matthew Tesoriero, Kirsty F. Smith, Shauna A. Murray

**Affiliations:** 1 University of Technology Sydney School of Life Sciences, Sydney, NSW, Australia; 2 Cawthron Institute, Nelson, New Zealand; Gujarat Institute of Desert Ecology, INDIA

## Abstract

A quantitative polymerase chain reaction (qPCR) assay was developed for the species-specific detection and quantification of *Gambierdiscus holmesii*, recently reported to produce known ciguatoxin (CTX) analogues in north Queensland. The assay was designed to target the ITS region and calibrated using synthetic gBlocks standards and cell-based standard curves to enable accurate quantification. The *G. holmesii*-specific qPCR assay demonstrated high specificity, efficiency, and sensitivity, with no cross-reactivity observed against closely related *Gambierdiscus* species. The assay was subsequently applied to environmental samples collected from Heron Island (Great Barrier Reef, Queensland), detecting *G. holmesii* in 5 out of 12 sites. This newly developed molecular tool enhances the capacity to monitor *G. holmesii* in the environment and supports improved surveillance of ciguatoxin-producing *Gambierdiscus* species in Australian waters, enabling earlier detection and more effective management of ciguatera poisoning risks to public health and fisheries.

## Introduction

Ciguatera poisoning (CP) is one of the most common non-bacterial seafood-borne illnesses, caused by the consumption of fish contaminated with ciguatoxins (CTXs), potent neurotoxins produced by benthic dinoflagellates from the genera *Gambierdiscus* and *Fukuyoa* [1]. CP is a significant public health issue in tropical and subtropical regions, including the Pacific, Caribbean, and Indian Oceans, where it poses serious risks to both human health and marine fisheries. Climate change may have further exacerbated the spread of both ciguatoxic fish and *Gambierdiscus* species into more temperate zones, raising concerns about the global expansion of CP [2]. The health impacts of CP are severe, encompassing a range of gastrointestinal, cardiovascular, and neurological symptoms that can persist over extended periods [3]. Compounding the issue, no universal treatment exists for CP, highlighting the critical need for effective monitoring and prevention strategies.

The causative toxins of CP, CTXs, bioaccumulate through marine food webs, particularly in herbivorous fish that graze on macroalgae colonised by the dinoflagellate producers [4]. Alongside CTXs, *Gambierdiscus* and *Fukuyoa* species also produce a suite of other toxins, such as maitotoxins (MTXs) [5–7], gambieric acids [8], gambierol [9], gambieroxide [10] and gambierone [11,12]. However, it is not clear yet if these compounds also play a role in CP [13], which further complicates detection and risk assessment.

Several species within the genus *Gambierdiscus* have demonstrated toxicity in different bioassays, including *G. australes*, *G. caribaeus*, *G. excentricus*, *G. pacificus*, *G. polynesiensis*, *G. toxicus*, *G. lapillus* and *G. holmesii* [14–23]. However, among these species, only *G. polynesiensis,* has repeatedly shown chemically detectable production of known CTX analogues when strains have been analysed using liquid chromatography mass spectrometry (LC-MS) [14,24]. The identification of known CTX- producing *Gambierdiscus* species directly in the field is critical for predicting and assessing the risk of CP outbreaks.

CP has been documented in Australia since the 18th century [25]. Over the years, significant outbreaks have occurred, including two human fatalities [26,27]. CP in Australia is predominantly associated with the consumption of reef fish from tropical waters along the east coast [26,28,29]. However, recent cases have also been linked to fish caught in more subtropical regions [30–32]. Despite the presence of CP in Australia, little is known about the CTX-producing *Gambierdiscus* species in this region, similar to many other places worldwide. Early efforts to isolate *Gambierdiscus* strains were made by Holmes, Lewis, and Gillespie [5,33,34]. However, the species identities from these studies remain unresolved, as they occurred prior to the current understanding of *Gambierdiscus* diversity and molecular taxonomy, and the morphological descriptions lack sufficient detail for species identification by modern standards [35]. Subsequent studies have provided some insights into the diversity of *Gambierdiscus* in tropical eastern Australia. Murray et al. (2014) [36] identified three species from the central Great Barrier Reef (GBR): *G. carpenteri*, a species resembling *G. belizeanus* (*G*. cf. *belizeanus*), and *Fukuyoa paulensis* (as *G*. cf. *yasumotoi*). Since then, *G. carpenteri* has been reported in the GBR [37] and as far south as Merimbula, a temperate location in southern New South Wales [38–40]. In addition, Kretzschmar et al. (2017) [41] described a novel species, *Gambierdiscus lapillus*, isolated from Heron Island in the southern GBR. More recently, another two species have been found and described: *Gambierdiscus lewisii* and *G. holmesii* [17]. These studies highlight the underexplored diversity of *Gambierdiscus* in Australian waters and the need for further investigations into their taxonomy and toxicology.

Traditional harmful algal monitoring approaches, such as the identification of species using light microscopy (LM) and scanning electron microscopy, can be used to differentiate between *Gambierdiscus* and other genera, but cannot always reach species-level identification and can be time-consuming and require highly specialised skills. Consequently, genetic sequencing has become essential for accurate identification [42], and molecular genetic assays are increasingly employed for this purpose.

Among these, quantitative polymerase chain reaction (qPCR) is a reliable and efficient method for the detection and quantification of *Gambierdiscus* cells. Several assays have been developed and used to detect and quantify cells: *G. belizeanus*, *G. caribaeus*, *G. carolinianus*, *G. carpenteri*, *G. ruetzleri* and *Gambierdiscus* ribotype 2 [43]; *G. australes*, *G. scabrosus, Gambierdiscus* sp. type 2 and *Gambierdiscus* sp. type 3 [44]; *Gambierdiscus/Fukuyoa* spp. and *F. paulensis* [45]; *G. polynesiensis* and *G. toxicus* [46]; *G. excentricus* and *G. silvae* [47]; *G. lapillus* [17] and *G. honu* [48]. Even though alternative approaches like isothermal DNA amplification, for example recombinase polymerase amplification (RPA) [49], offer rapid and energy-efficient results, qPCR is preferred due to its sensitivity, specificity, and established protocols. The development of qPCR assays addresses many of the limitations of microscopy methods, particularly in differentiating between species of *Gambierdiscus*, including identifying potential ciguatoxin-producing species. Molecular approaches also offer the ability to screen environmental samples for potential CP risk hotspots, significantly improving the timeliness and accuracy of monitoring programs. The introduction of field-deployable molecular diagnostic tools, such as portable qPCR devices, represents a transformative shift in the management of CP and HABs. These tools allow for the real-time detection of toxic *Gambierdiscus* species directly in marine environments, reducing the need for laboratory-based testing and enabling faster responses to emerging risks. By enabling species-specific identification and quantification, these methods provide vital data for managing CP risks as the distribution of toxin producing species of *Gambierdiscus* representing a significant step forward in environmental monitoring and helping CP risk management by offering timely, accurate, and accessible solutions to protect public health and marine ecosystems.

Recently, a strain of *Gambierdiscus holmesii* from north Queensland was shown for the first time to producer known analogues of CTXs [24]. This highlighted a new, uncharacterised risk of ciguatoxins in regions of north Queensland. Therefore, it has become clear that there is an urgent need to be able to identify and quantify this species. In this study, a qPCR assay was developed specifically for the detection and quantification of *G. holmesii* in environmental samples. This assay showed the potential to be used as a valuable tool for monitoring *G. holmesii* populations in endemic regions where the risk of CP represents a threat for human health.

## Materials and methods

### Field sampling

**Fig 1:**
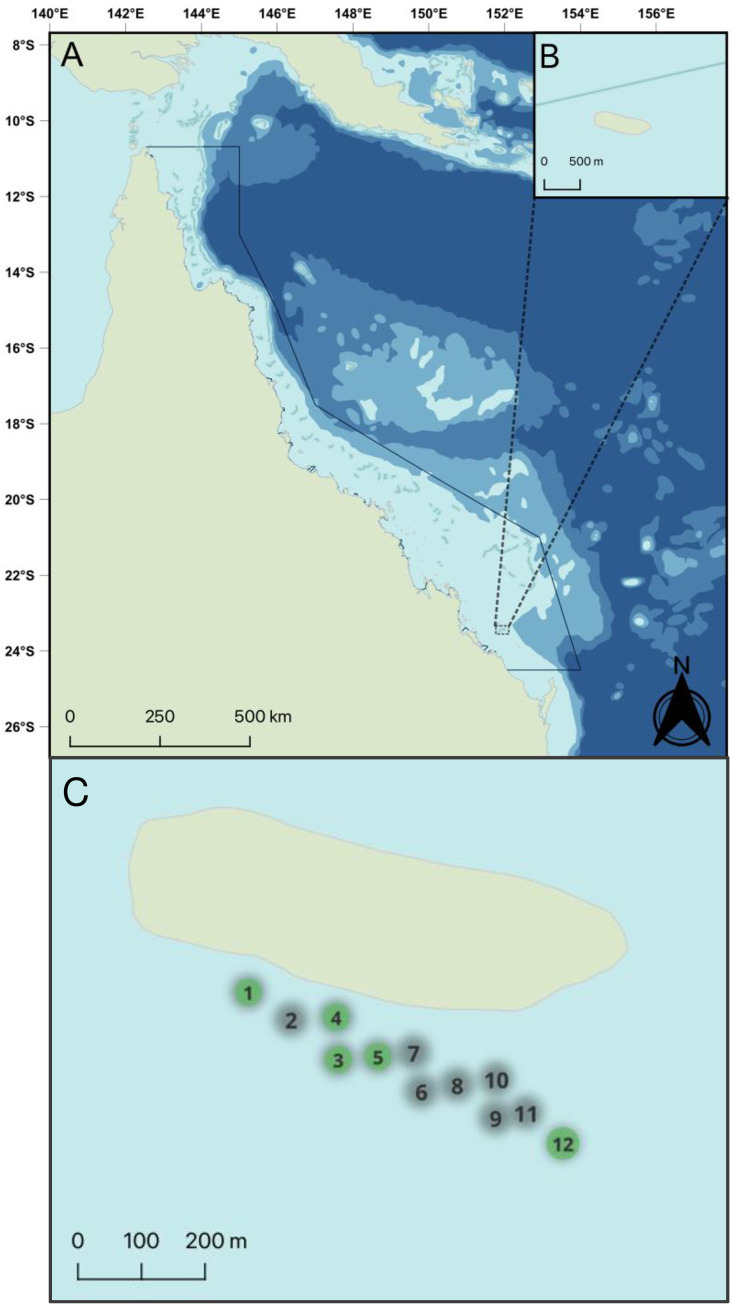
Heron Island location (A and B) and sampling locations. Sampling points where *G. holmesii* presence was detected are highlighted in green (C). Three replicates were collected at each site. Map realized in QGIS 3.42.1-Münster by Greta Gaiani.

**Fig 2:**
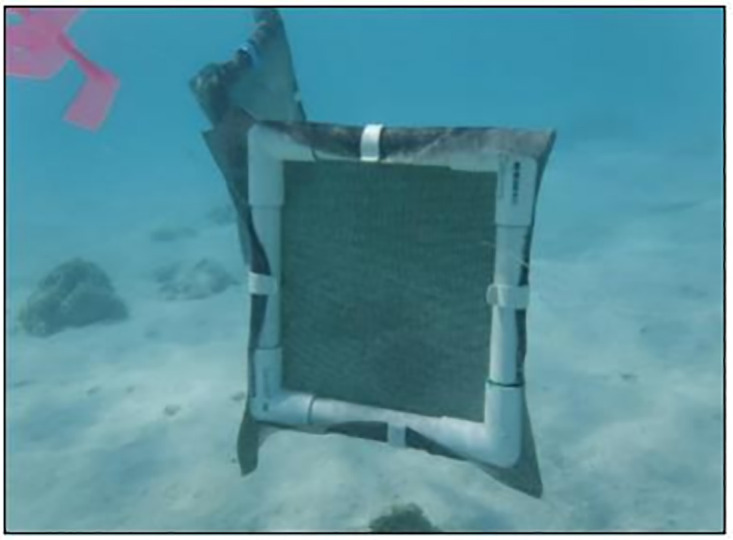
Example of mesh artificial substrate (MAS) sampling technique. Photo by Caroline Dornelles de Azevedo.

Environmental surveys were conducted at twelve sites around Heron Island (23° 26′ 18.7″ S, 151° 54′ 30.2″ E) from 7–17 March 2018 (Fig 1). Samples were collected from 12 sites and triplicates were obtained by placing three mesh artificial substrates (MAS) samplers per site (Fig 2). MAS were rectangular fiberglass screens (21 × 21 cm; mesh size 1–3 mm) mounted onto rigid frames using a clip-in system (SuperFrame©). Each frame was fitted with a weight and a small subsurface float to maintain the device at ~0.5 m depth (Fig 2). This configuration ensured the screens were oriented perpendicularly to the prevailing current, facilitating water flow across the mesh surface and maximizing attachment of benthic dinoflagellates.

Screens were incubated in situ for 24 h to optimize collection of *Gambierdiscus* cells. Microalgal cells attached to artificial mesh substrates were collected following gentle rinsing with seawater to remove debris. Each mesh was then transferred into a sterile container containing 20–50 mL of seawater, and cells were detached by mild agitation using manual shaking. Both sides of the mesh were subsequently flushed with a wide-bore pipette to ensure complete detachment. The mesh was then removed, rinsed again, and the combined suspensions were collected into a single sample. Cell abundances were normalized to the effective surface area of the substrate and expressed as cells cm^-2^. Following retrieval, each sample was stabilized with 200 µL Longmire’s buffer (100 mM Tris-HCl, 100 mM EDTA, 10 mM NaCl, 0.5% SDS, 0.2% sodium azide) and stored at –20 °C until analysis at the University of Technology Sydney (UTS).

Field sampling was conducted under a Letter of Authorisation for Limited Impact Research in the Great Barrier Reef Marine Park (Ref. RES17/1068), issued by the University of Technology Sydney Research and Innovation Office under the GBRMPA accreditation framework for limited-impact research. Access to Heron Island Research Station was approved by the station management.

### Culturing and DNA extraction

*Gambierdiscus holmesii* strains (UTSHI6B1, OIRS406 and HG5) were obtained from samples collected on Heron Island [17,24,39,41] during different sampling campaigns in 2018. In these studies, cell cultures were obtained as described in [17,24,39]. Briefly, single cells of *G. holmesii* were isolated under an inverted light microscope (Nikon Eclipse TS100, Japan), using the micropipette technique [50], washed three times in sterile seawater, and transferred into modified K medium [39]or f/10 medium [17,24]. Species identification was confirmed through Sanger sequencing, GenBank accession numbers are listed in Table 1. Strain UTSHI6B1 was incubated at 20 °C under ~100 µmol photons m^−^² s^−^¹ with a 12:12 h light:dark cycle [17]. Strain OIRS406 was maintained at 25 °C under ~100 µmol photons m^−^² s^−^¹ with a 14:10 h light:dark cycle [24], whereas strain HG5 was cultured at 27 °C under ~60 µmol photons m^−^² s^−^¹ with a 12:12 h light:dark cycle [39]. Strains were sub-cultured every 3 weeks.

**Table 1:** Sequences of *Gambierdiscus* spp. used in the alignments to design the quantitative PCR primers.

Species name	Strain number	GenBank accession number	Origin
*G. holmesii*	HG5	MH790454	Heron Island (Australia)
*G. holmesii*	UTSHI6B1	MH790456	Heron Island (Australia)
*G. polynesiensis*	TB92	MT040032	Tubuai (French Polynesia)
*G. silvae*	VGO1180	MH790453	Canary Islands (Spain)
*G. jejuensis*	C-1	AB499538	Jeju Island (Korea)
*G. caribaeus*	GCJJ1_	HE775087	Florida Keys (USA)
*G. carpenteri*	NOAA1	GU968493	Gulf of Mexico (USA)
*G. belizeanus*	NOAA17	GU968497	Belize Barrier Reef (Caribbean)
*G. scabrosus*	A-1	AB499593	Yaeyama Island (Japan)

In this work, laboratory cultures of UTSHI6B1, OIRS406 and HG5 were established to obtain cell pellets for the assay optimization. Cell were counted every 3−4 days using a Sedgewick rafter chamber. When cell reached approximately 1,200 cell mL ^−1^ (about 21 days for OIRS406 and 25 days for UTSHI6B1 and HG5), DNA was extracted using the DNeasy PowerSoil Pro Kit (Qiagen®) as per the manufacturer’s protocol. The concentration and purity of the extracted genomic DNA was measured using a Nanodrop instrument and a Qubit 2.0 Fluorometer (Nanodrop2000 – Qubit 2.0 Fluorometer – ThermoFisher Scientific®, Massachusetts, USA), and the integrity of the DNA was visualised on 1% agarose gel.

For the DNA extraction of the environmental samples was performed as follow: samples were centrifuged at 3,200 × g for 10 min, the supernatant was discarded and the pellet resuspended in sterile seawater. Up to 500 µL were processed with a QIACUBE HT (QIAGEN®) instrument using the QUIACUBE DNA extraction kit and following manufacturer’s instructions. Extracts were used for downstream qPCR analysis with the selected primers for *G. holmesii* detection. Each extraction was performed in triplicates.

### Primer design

Primer sets specific for *G. holmesii* were designed within the internal transcribed spacer (ITS) region of the ribosomal DNA. To encompass sequence variability and assess primer specificity, we aligned all *G. holmesii* ITS sequences available in GenBank with representative ITS sequences from *Gambierdiscus* species present in Australian waters (*G. polynesiensis*, *G. carpenteri*) and from closely related species not reported in Australia (*G. silvae*, *G. jejuensis*, *G. caribaeus*, *G. belizeanus*, and *G. scabrosus*). At first, conserved regions of the alignment specific to *G. holmesii* were identified manually, and primers were tested first *in silico* through Geneious software, v8.1.7, using MUSCLE algorithm parameters [51]. Then, selected primers were synthesised by Integrated DNA Technologies (USA) and tested via qPCR against the available *Gambierdiscus* species in UTS culture collection. Accession numbers for each strain are listed in Table 1. The qPCR assays were performed in a total volume of 5 μL containing 2.5 μL SYBR Select Master Mix (Thermo Fisher Scientific, Australia), 0.2 μM forward and reverse primers, 1.1 μL MilliQ water, and 1 μL DNA template. Cycling conditions consisted of 3 min at 95 °C, then 39 cycles of 95 °C for 15 seconds and 60 °C for 30 seconds, followed by a temperature gradient tested from 55 °C (0.05 seconds) to 95 °C for melting curve construction. Each qPCR run included a no-template control (NTC) and a positive control consisting of genomic DNA from *G. holmesii*. All samples, standards and controls were analysed in triplicate qPCR reactions. The qPCR equipment utilized in the experiment was BioRad CFX 96 Real Time System®. Cq values were determined automatically using Bio-Rad CFX Manager Software v3.1.

### Evaluation of primer specificity and sensitivity

**Fig 3:**
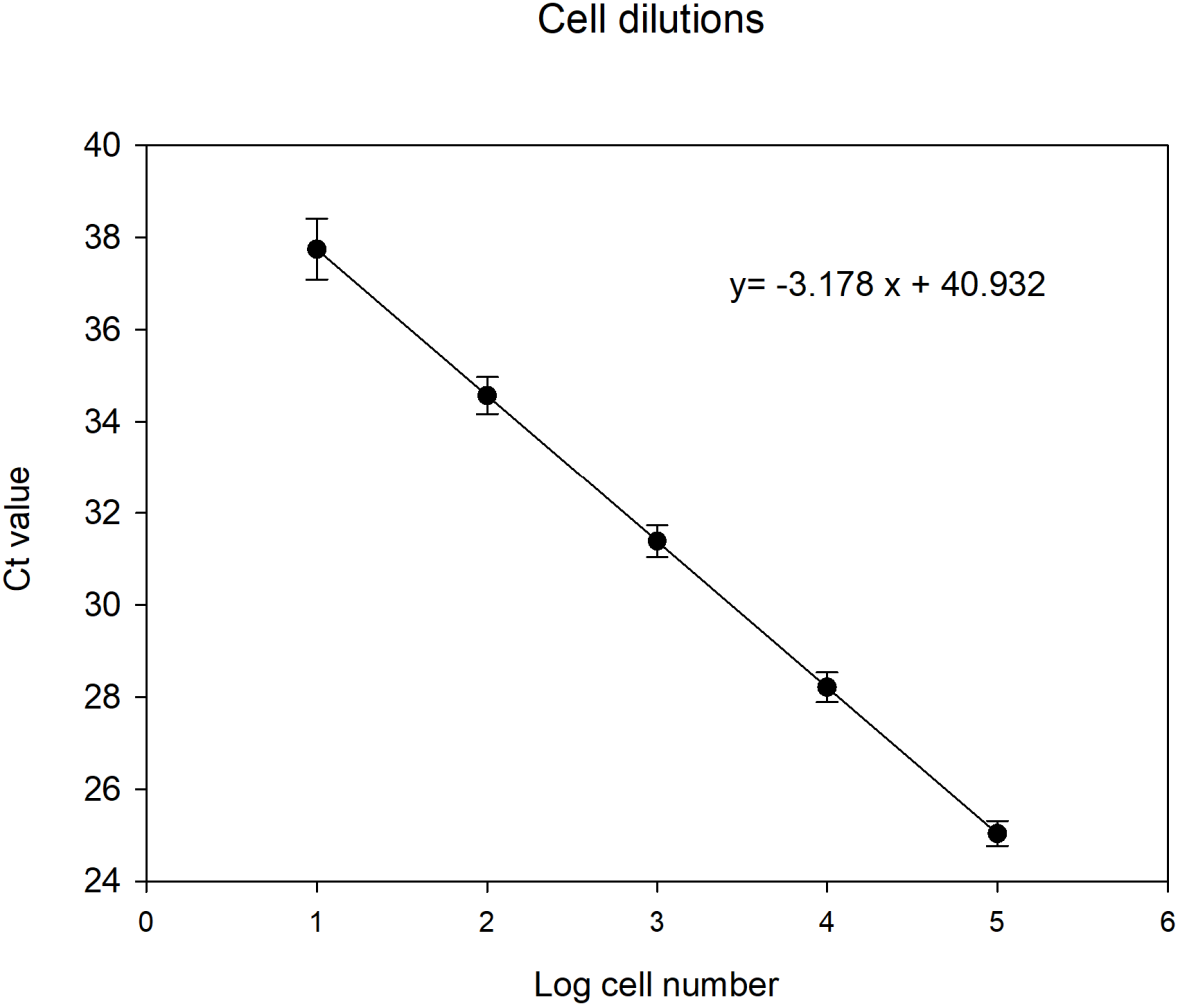
Quantitative PCR cell-based standard curve of a strain of *Gambierdiscus holmesii* OIRS406.

Primer sensitivity was assessed by qPCR under the conditions listed above. Primer specificity was assessed by (i) in silico sequence alignment, (ii) amplification of target and non-target *Gambierdiscus* species, and (iii) melt curve analysis. A single, distinct melt curve peak was used to confirm amplification of a unique PCR product. Various calibration curves were generated to define the assay’s quantitative detection range. At first, Cell-based standard curves were prepared from genomic DNA of *G. holmesii* strain OIRS406. Cell-based standard curves were prepared from genomic DNA extracted from *G. holmesii* strain OIRS406. DNA extracts corresponding to 5,000 cells were serially diluted 10-fold to generate standards ranging from 5,000 to 0.05 cell equivalents per reaction. These standards were used to construct the cell-based standard curve shown in Fig 3. Reactions were run in triplicate on a StepOnePlus™ Real-Time PCR System (Applied Biosystems, Thermo Fisher Scientific, Waltham, MA, USA). Then, qPCR assay efficiency was evaluated using gBlocks (Integrated DNA Technologies®, Illinois, USA). The molecular weight and concentration of the synthesised gene fragments were provided by IDT Technologies® and used to accurately determine the number of gene copies per µL. The lyophilised gBlocks fragments were reconstituted in 1x TE buffer (Tris 1M, EDTA 0.5, pH = 8) to achieve a final concentration of 10^8^ copies. Serial dilutions ranging from 10^8^ to 10^2^ were tested in triplicate using a StepOnePlus™ Real-Time PCR System (Applied Biosystems, Thermo Fisher Scientific, Waltham, MA, USA). To determine the number of genomic DNA copies of the target ITS region per cell, Cq values from cell-based standards were converted to gene copies per reaction using a gBlocks calibration curve, and gene copies per cell were then calculated by dividing the estimated gene copies by the known number of cells in each standard. A completed MIQE (Minimum Information for Publication of Quantitative Real-Time PCR Experiments) checklist is provided as Supplementary S1 Table.

## Results

### Evaluation of primer specificity and sensitivity

The 277F -343R primer pair presented a standard curve slope of -3.178, corresponding to an amplification efficiency of 106.3% (Table 2, Fig 3) and produced an amplified product of 143 bp for *G. holmesii* strain OIRS406, with a single melt curve peak of 80 °C. No signal for primer dimers or sample contamination was detected. The primer pair designed in this study did not present amplification for any non-target *Gambierdiscus* species tested (Table 1), except for *G. polynesiensis* which presented with a different melting curve at 78.5°C and *G. silvae*, which presented two small peaks for the melting curve temperature (one at 76 °C and another at 86 °C) (S1 Fig). In both cases the reported melting curves were over the 0.5 °C melting temperature shift threshold for species-specific amplification established by Díaz-Asencio et al. (2019) [52].

**Table 2:** *Gambierdiscus holmesii* quantitative PCR primer sets targeting the internal transcribed spacers regions (ITS) of the ribosomal DNA used in this study.

Assay component	Sequence (5’-3’)
277 F	TTG AAG TGA GGT GGG TGT GG
343 R	TGT GTG CAT GGC AGT TTT GG
gBlock_*G. holmesii*	ACT GTG GTG CAC AAC TTG ACT TGC AGT ACT CCG TGA ATT CTT GAC AAC TGA ATG CCT GAT GCA CCC TTG GGA TAG TCC GAA GGG TGT GTC CAG TTT GAA GTG AGG TGG GTG TGG GCC ACA ATG AAT GTG AAG GTG TGC ACT TGT GTG CAT GGC AGT TTT GGC TGG CCA GGT GCA CTT TAG TGC AGG CTC TGT GCG CCG GTG GTG TGG GCA CCT CTT GGC CGT GGC GTG CTG GCA

**Fig 4:**
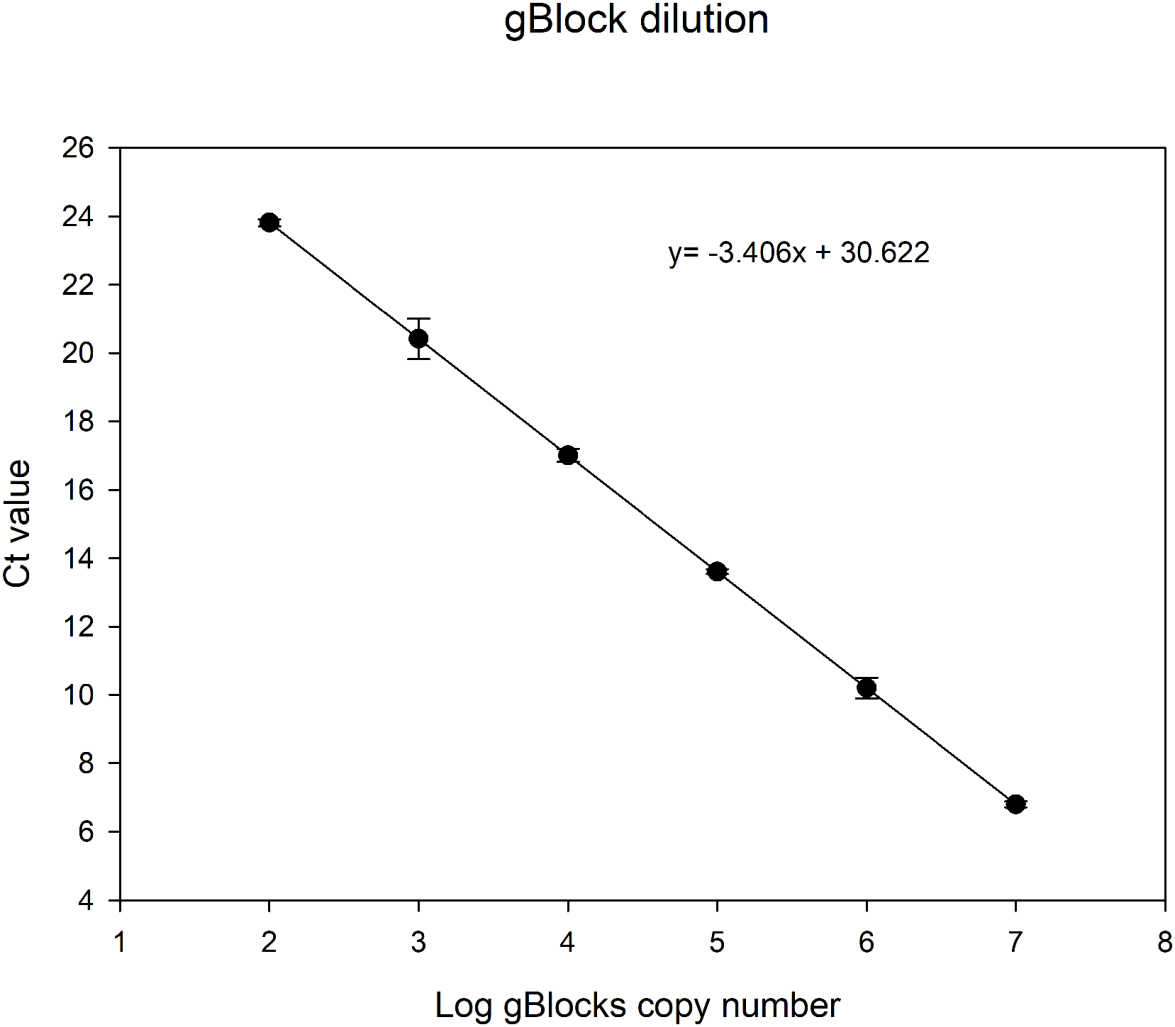
Quantitative PCR gBlock- based standard curve of *Gambierdiscus holmesii.*

The qPCR assay (primers 277 F and 343 R) developed for *G. holmesii* demonstrated high sensitivity and specificity. A standard curve was generated from serial dilutions of *G. holmesii* gBlocks fragments (Fig 4), yielding a slope of -3.406, an amplification efficiency of 95%, and a strong linear relationship (R² > 0.99) between Cq values and the log of gBlocks fragments. No significant off-target amplification was observed when testing the assay against closely related *Gambierdiscus* species, such as *G. polynesiensis* and *G. silvae*, confirming the assay’s high specificity. The Limit of detection (LOD) was defined as the lowest gBlock input detected in ≥95% of replicate reactions with correct amplicon identity and no negative control (no template control) amplification. Using 8–12 replicates per level across two runs, the LOD was 2 copies per reaction, highlighting its ability to detect *G. holmesii* at minimal concentrations.

### Environmental samples

The qPCR assay targeting *G. holmesii* designed in this study was used to screen 12 samples collected in the southern part of Heron Island (Fig 1). The species was detected at 5 sites and only in site number 5 was it detected in all the replicates (Fig 1, Table 3). No Cq value was detected for all the NTCs. Cell number were estimates using the assay standard curves. Presence of *G. holmesii* in the area was confirmed using different techniques in previous studies [17,24] from our group conducted at Heron Island at the same time as this study. Particularly, OIRS406 was isolated from Sample 1 (Fig 1) and its morphological and morphometric description are reported in Murray et al., 2025 [24]. In the same study, presence of *G. holmesii* was detected with metabarcoding. Melting curves from positive signals were checked, to ensure specificity. Any positive result with a different melting curve than the one specific for *G. holmesii* has been excluded from the analysis.

**Table 3:** Sampling sites and quantitative PCR-based quantification of *Gambierdiscus holmesii*. Dash (–) indicates no detection.

Site	Coordinates	Replicate	Cells of *G. holmesii* per 100 cm^2^
1	23°26’36.84“ S, 151°54’47.18” E	A	189
		B	127
		C	–
2	23°26’38.55“ S, 151°54’49.55” E	A	–
		B	–
		C	–
3	23°26’40.57“ S, 151°54’52.16” E	A	637
		B	–
		C	398
4	23°26’38.62“ S, 151°54’52.04” E	A	–
		B	–
		C	88
5	23°26’40.40“ S, 151°54’54.36” E	A	679
		B	1071
		C	321
6	23°26’42.32“ S, 151°54’56.76” E	A	–
		B	–
		C	–
7	23°26’40.18“ S, 151°54’56.33” E	A	–
		B	–
		C	–
8	23°26’41.98“ S, 151°54’58.74” E	A	–
		B	–
		C	–
9	23°26’43.79“ S, 151°55’00.87” E	A	–
		B	–
		C	–
10	23°26’41.65“ S, 151°55’00.91” E	A	–
		B	–
		C	–
11	23°26’43.51“ S, 151°55’02.56” E	A	–
		B	–
		C	–
12	23°26’45.22“ S, 151°55’04.59” E	A	42
		B	–
		C	–

## Discussion

This study describes a species-specific qPCR assay for *Gambierdiscus holmesii* with performance characteristics suitable for quantitative field applications. The primers showed high specificity, with no amplification against other *Gambierdiscus* species except *G. polynesiensis* and *G. silvae*. However, distinct melt-curve peaks (80 °C for *G. holmesii*, 78.5 °C for *G. polynesiensis*, 76 °C and 86 °C for *G. silvae*) allow reliable discrimination. The differences exceed the ± 0.5 °C melt-temperature shift threshold for species-specific amplification [52]. Standard curves exhibited high linearity and efficiency within commonly accepted ranges for robust qPCR assays. Together, these results indicate that the *G. holmesii* assay can reliably discriminate targets in mixed assemblages typical of reef substrates.

Because CP risk is linked to *Gambierdiscus* abundance [17] and *G. holmesii* is one of the two species with LC-MS–confirmed CTX production in the Pacific region [24], defining copy number per cell is important for quantitative inference. We converted cell-standard Cq values to copies per reaction using the gBlocks calibration curve on linear scales, yielding an rDNA copy number of 9.59 × 10⁴ copies cell^−^¹. This aligns with estimates for other species (Vandersea et al., 2012 [43]: 6.9 × 10² and 2.15 × 10⁴ for *G. belizeanus* and *G. caribaeus* respectively; Kretzschmar et al., 2019 [17]: 5.9 × 10^3^ to 2.24 × 10^4^ for *G. lapillus*) but is lower than values reported by Nishimura et al. (2016) [44] (5.32 × 10^5^ for *G. scabrosus*; 2.26 × 10⁶ for *G.* sp. type 3). Discrepancies may reflect inter and intraspecific differences in cell size and genomic DNA [17,49], underestimation from “ghost cells” lacking amplifiable DNA [44,53], and methodological factors (extraction chemistry, primer affinity). Genuine variation in rDNA content is also plausible, as shown by species/strain differences at the same DNA concentration [49]. Large intraspecific ranges have also been observed *Alexandrium* studies [54–56]. Given these uncertainties, probe-based qPCR or ddPCR can improve specificity and accuracy for copies-per-cell calibration, although at higher cost.

The assay detected *G. holmesii* at 5/12 sites on Heron Island (Fig 1, Table 3), with higher occurrence in the southern sector, consistent with metabarcoding results [24]. Absence at several sites likely reflects the strong small-scale patchiness of *Gambierdiscus* [17] and its facultative epiphytic behaviour observed in culture. Spatial patterns also contrasted with *G. lapillus*, which was more abundant on the island’s eastern side [17]. Methodological differences may contribute: this study used mesh artificial substrates (MAS), whereas Kretzschmar et al. [17] and Murray et al. [24] sampled macroalgae. The presence of *G. holmesii* in the area was confirmed using different techniques described in a previous work [24] carried out by our team in Heron Island at the same time as this study. In that study [24], *G. holmesii* was detected also in outer-reef locations. We therefore hypothesise that macroalgae may provide greater sensitivity for detecting benthic taxa because they integrate established epiphytic communities, whereas MAS deployed for 24 h capture only organisms that colonise the substrate during that period.

Our MAS abundances (42–1070 cells 100 cm^−^²) are comparable to values reported from Belize [57] (~211 cells 100 cm^−^²), the Florida Keys [58] (~20–1848 cells 100 cm^−^²) and St. Thomas [58] (~112 cells 100 cm^−^²), but lower than Malaysia [57] (566–1349 cells 100 cm^−^²), where the sampling was conducted in a strong tidal disrupting settlement. MAS offers a standardized surface-area basis, removes host-specific biases, and can be useful in hydrodynamically variable settings by providing a consistent attachment surface [57]. However, it requires two site visits and may under-represent host-preference dynamics that influence toxin transfer through food webs [1,58]. Accordingly, MAS is best used as a complement (not a replacement) to macroalgal sampling when mapping *Gambierdiscus* distributions and interpreting ecological risk.

Some *Gambierdiscus* species show preferences for particular macroalgal hosts [59,60], potentially due to benefits such as attachment substrate, protection from turbulence, shading, and access to organic compounds [1]. Although dinoflagellates often prefer calm, stable environments [59,61,62], *Gambierdiscus* has also been recorded at wave-exposed sites (Mayotte [63], Hawai‘i [64], Canary Islands [15]). Small-scale turbulence can promote rapid attachment [65]. Based on our results and prior work, *G. holmesii* appears most frequent in sheltered, low-energy habitats (Heron, Orpheus, Beaver Reef, Vava’u; Murray et al., 2025 [24]; this study) and commonly co-occurs with *G. lapillus* [17] and *G. polynesiensis* [24].

Our *Gambierdiscus holmesii* abundances on artificial substrates ranged from 42 to 1,000 cells 100 cm^−^². Applying the artificial-substrate–to–macroalga conversion of Tester et al. (2014) [57] yields an estimated 8–190 cells g^−^¹ wet weight. These values overlap the low end of *G. lapillus* reported from Australia [17,44] (~5 cells g^−^¹ ww) but are noticeably lower than *G. polynesiensis* densities reported from French Polynesia, where qPCR on window screens measured 1,225–38,300 cells 150 cm^−^² [46]. For unit comparability, our range corresponds to approximately 63–1,500 cells 150 cm^−^². However, cell density alone does not reliably predict toxin levels [66]. A prevailing view is that toxicity in nature is driven by co-occurring toxic species with variable contributions [42,46,52,61,67]. Less-toxic species may still contribute to CTX bioaccumulation through long-term persistence [68], although depuration of toxins from fish may also mean that low level CTX producers do not contribute overly to the CTX load [24]. Microbial interactions (e.g., quorum-sensing bacteria) may modulate growth and toxin output [69]. Overall, toxinogenesis reflects interacting genetic, physiological, environmental and microbial drivers [14,19,32,70–75]. Therefore, long-term ecological surveys may be critical to track population cycles, identify conditions that favour blooms, and link them to CTX-like toxicity and ciguatera risk, yet such studies remain scarce.

Conducting long-term surveys requires sustained funding and coordination across years and sites. These demands, together with the spatiotemporal patchiness of *Gambierdiscus*, help explain why such datasets are uncommon. In contrast, molecular methods such as qPCR and ddPCR provide a more rapid, sensitive, and scalable approach. These tools allow for the detection of species at low abundances, yield species-specific resolution, and enable standardized comparisons across studies. They can also be applied to archived samples, reducing the need for continuous on-site monitoring. Together, these advantages make molecular approaches a powerful and efficient complement to classical long-term surveys, supporting the development of monitoring frameworks that are both cost-effective and capable of capturing the ecological dynamics of *Gambierdiscus* populations over time.

This study establishes a reliable qPCR assay for detecting *G. holmesii* in natural samples. To ensure accuracy, future work should validate absolute quantification with ddPCR and expand field trials across seasons, regions, and host substrates. Linking molecular abundance data with toxin measurements, together with metabarcoding and transcriptomic approaches, will enable a shift from simple presence/absence observations toward more meaningful assessments of ciguatera risk. Collectively, these approaches will enhance our understanding of *Gambierdiscus* dynamics and toxicity mechanisms and strengthen monitoring frameworks in vulnerable regions.

## Conclusion

We developed and validated a species-specific qPCR assay for *Gambierdiscus holmesii* and showed that it performs well on environmental samples from Heron Island, an island of the Great Barrier Reef in Queensland, Australia. The assay is sensitive, specific, and suitable for routine use, making it a practical addition to CP monitoring and early-warning efforts where multiple *Gambierdiscus* species co-occur. Used alongside an appropriate field sampling method and paired with toxin measurements, the assay can move programs beyond simple presence/absence toward risk-relevant interpretation.

In future, further application of this method across seasons, sites, and host substrates, and integrating qPCR outputs with toxin data and community profiling (e.g., metabarcoding or FISH) will improve links between abundance and risk. Overall, this assay offers a scalable, cost-efficient tool for targeted surveillance of CTX-producing species and provides a practical foundation for ciguatera risk assessment and management in vulnerable reef systems.

## Supporting information

S1 Fig Melting curve amplification plots. Plots were obtained from screening with the G. holmesii primers designed in this study. Distinct melting peaks were observed for (a) *G. holmesii* (80 °C), (b) *G. polynesiensis* (78.5 °C), and (c) ***G. silvae*** (78 °C and 87 °C), enabling clear species discrimination.
(PDF)

S1 Table Completed MIQE (Minimum Information for Publication of Quantitative Real-Time PCR Experiments) checklist for the development and validation of the *Gambierdiscus holmesii* qPCR assay. (PDF)
