# Supplementary figures and images for: Development of a quantitative PCR assay to detect Gambierdiscus holmesii, a ciguatoxin producing species from Australian waters
Source: PLoS One. 2026 Aug 3;21(8):e0355213. doi: 10.1371/journal.pone.0355213 (PMC13432126; doi:10.1371/journal.pone.0355213)

Melt Peak

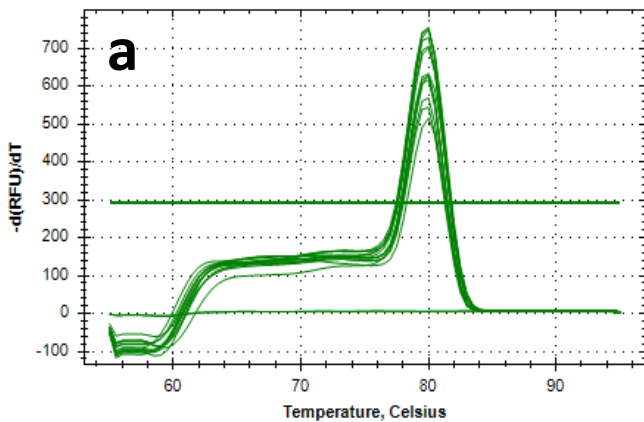

Melt Peak

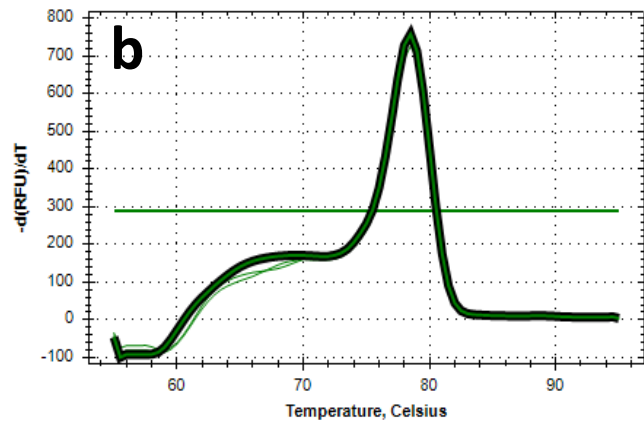

Melt Peak

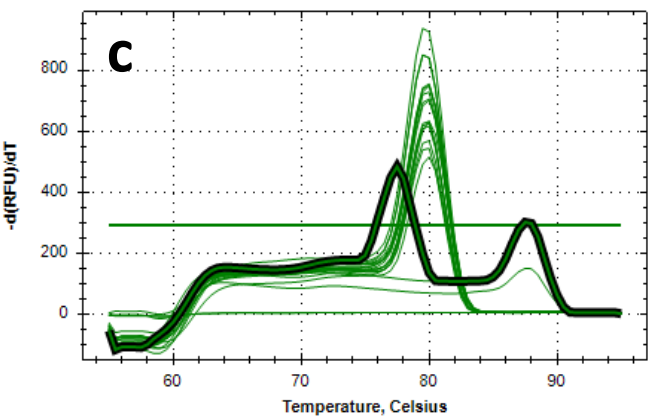

Supplement: S1 Fig — Plots were obtained from screening with the G. holmesii primers designed in this study. Distinct melting peaks were observed for (a) G. holmesii (80 °C), (b) G. polynesiensis (78.5 °C), and (c) G. silvae (78 °C and 87 °C), enabling clear species discrimination. (PDF) [file pone.0355213.s001.pdf]
