# Supplementary material for: Development of a quantitative PCR assay to detect Gambierdiscus holmesii, a ciguatoxin producing species from Australian waters
Source: PLoS One. 2026 Aug 3;21(8):e0355213. doi: 10.1371/journal.pone.0355213 (PMC13432126; doi:10.1371/journal.pone.0355213)
Supplement: S1 Table — (PDF) [file pone.0355213.s002.pdf]

ITEM TO CHECK	IMPORTANCE	CHECKLIST
**EXPERIMENTAL DESIGN**		
Definition of experimental and control groups	**E**	Reported (Methods).
Number within each group	**E**	Reported (Methods).
Assay carried out by core lab or investigator's lab?	D	Investigator's laboratory (UTS).
Acknowledgement of authors' contributions	D	Reported (Authors contribution)
**SAMPLE**		
Description	**E**	Reported (Methods).
Volume/mass of sample processed	D	Reported (Methods).
Microdissection or macrodissection	**E**	N/A
Processing procedure	**E**	Reported (Methods).
If frozen - how and how quickly?	**E**	Reported (Methods).
If fixed - with what, how quickly?	**E**	Reported (Methods).
Sample storage conditions and duration (especially for FFPE samples)	**E**	Reported (Methods).
**NUCLEIC ACID EXTRACTION**		
Procedure and/or instrumentation	**E**	Reported (Methods).
Name of kit and details of any modifications	**E**	Reported (Methods).
Source of additional reagents used	D	N/A
Details of DNase or RNAse treatment	**E**	N/A
Contamination assessment (DNA or RNA)	**E**	Reported (Methods and Results)
Nucleic acid quantification	**E**	Reported (Methods)
Instrument and method	**E**	Reported (Methods)
Purity (A260/A280)	D	Not reported
Yield	D	Not reported
RNA integrity method/instrument	**E**	N/A
RIN/RQI or Cq of 3' and 5' transcripts	**E**	N/A
Electrophoresis traces	D	N/A
Inhibition testing (Cq dilutions, spike or other)	**E**	Not performed
**REVERSE TRANSCRIPTION**		
Complete reaction conditions	**E**	N/A
Amount of RNA and reaction volume	**E**	N/A

Priming oligonucleotide (if using GSP) and concentration	**E**	N/A
Reverse transcriptase and concentration	**E**	N/A
Temperature and time	**E**	N/A
Manufacturer of reagents and catalogue numbers	D	N/A
Cqs with and without RT	D	N/A
Storage conditions of cDNA	D	N/A
**qPCR TARGET INFORMATION**		
If multiplex, efficiency and LOD of each assay.	**E**	N/A
Sequence accession number	**E**	Reported (Table 1)
Location of amplicon	D	Not reported
Amplicon length	**E**	Reported (Results)
In silico specificity screen (BLAST, etc)	**E**	Reported (Methods)
Pseudogenes, retropseudogenes or other homologs?	D	N/A
Sequence alignment	D	Not reported
Secondary structure analysis of amplicon	D	N/A
Location of each primer by exon or intron (if applicable)	**E**	N/A
What splice variants are targeted?	**E**	N/A
**qPCR OLIGONUCLEOTIDES**		
Primer sequences	**E**	Reported (Table 2)
RTPrimerDB Identification Number	D	N/A
Probe sequences	D	N/A
Location and identity of any modifications	**E**	N/A
Manufacturer of oligonucleotides	D	Reported (Methods)
Purification method	D	Not reported
**qPCR PROTOCOL**		
Complete reaction conditions	**E**	Reported (Methods)
Reaction volume and amount of cDNA/DNA	**E**	Reported (Methods)
Primer, (probe), Mg++ and dNTP concentrations	**E**	Reported (Methods)
Polymerase identity and concentration	**E**	Reported master mix (Methods)
Buffer/kit identity and manufacturer	**E**	Reported (Methods)
Exact chemical constitution of the buffer	D	N/A
Additives (SYBR Green I, DMSO, etc.)	**E**	Reported (Methods)

Manufacturer of plates/tubes and catalog number	D	Not reported
Complete thermocycling parameters	**E**	Reported (Methods)
Reaction setup (manual/robotic)	D	Not reported
Manufacturer of qPCR instrument	**E**	Reported (Methods)
**qPCR VALIDATION**		
Evidence of optimisation (from gradients)	D	Not reported
Specificity (gel, sequence, melt, or digest)	**E**	Reported (Results)
For SYBR Green I, Cq of the NTC	**E**	Reported (Results)
Standard curves with slope and y-intercept	**E**	Reported (Figure 3 and 4)
PCR efficiency calculated from slope	**E**	Reported (Results)
Confidence interval for PCR efficiency or standard error	D	Not reported
r2 of standard curve	**E**	Reported (Results)
Linear dynamic range	**E**	Reported (Figure 3 and 4)
Cq variation at lower limit	**E**	Not determined
Confidence intervals throughout range	D	Not determined
Evidence for limit of detection	**E**	Reported (Results)
If multiplex, efficiency and LOD of each assay.	**E**	N/A
**DATA ANALYSIS**		
qPCR analysis program (source, version)	**E**	Reported (Methods)
Cq method determination	**E**	Reported (Methods)
Outlier identification and disposition	**E**	N/A
Results of NTCs	**E**	Reported (Results)
Justification of number and choice of reference genes	**E**	N/A
Description of normalisation method	**E**	N/A
Number and concordance of biological replicates	D	Reported (Table 3)
Number and stage (RT or qPCR) of technical replicates	**E**	Reported (Results)
Repeatability (intra-assay variation)	E	Not reported (technical triplicates performed)
Reproducibility (inter-assay variation, %CV)	D	Not determined
Power analysis	D	N/A
Statistical methods for result significance	**E**	N/A - no statistical hypothesis testing performed
Software (source, version)	E	Reported (Methods)

Cq or raw data submission using RDML	**D**	Not submitted
